# Adherence to the Standards for Reporting of Diagnostic Accuracy (STARD) 2015 Guidelines in Acute Point-of-Care Ultrasound Research

**DOI:** 10.1001/jamanetworkopen.2020.3871

**Published:** 2020-05-01

**Authors:** Ross Prager, Joshua Bowdridge, Hashim Kareemi, Chris Wright, Trevor A. McGrath, Matthew D. F. McInnes

**Affiliations:** 1 Department of Medicine, University of Ottawa, Ottawa, Ontario, Canada; 2 Department of Emergency Medicine, University of Ottawa, Ottawa, Ontario, Canada; 3 Department of Radiology, University of Calgary, Calgary, Alberta, Canada; 4 Department of Radiology, University of Ottawa, Ottawa, Ontario, Canada; 5 Clinical Epidemiology Program, Ottawa Hospital Research Institute, The Ottawa Hospital, Ottawa, Ontario, Canada

## Abstract

**Importance:**

Incomplete reporting of diagnostic accuracy research impairs assessment of risk of bias and limits generalizability. Point-of-care ultrasound has become an important diagnostic tool for acute care physicians, but studies assessing its use are of varying methodological quality.

**Objective:**

To assess adherence to the Standards for Reporting of Diagnostic Accuracy (STARD) 2015 guidelines in the literature on acute care point-of-care ultrasound.

**Evidence Review:**

MEDLINE was searched to identify diagnostic accuracy studies assessing point-of-care ultrasound published in critical care, emergency medicine, or anesthesia journals from 2016 to 2019. Studies were evaluated for adherence to the STARD 2015 guidelines, with the following variables analyzed: journal, country, STARD citation, STARD-adopting journal, impact factor, patient population, use of supplemental material, and body region. Data analysis was performed in November 2019.

**Findings:**

Seventy-four studies were included in this systematic review for assessment. Overall adherence to STARD was moderate, with 66% (mean [SD], 19.7 [2.9] of 30 items) of STARD items reported. Items pertaining to imaging specifications, patient population, and readers of the index test were frequently reported (>66% of studies). Items pertaining to blinding of readers to clinical data and to the index or reference standard, analysis of heterogeneity, indeterminate and missing data, and time intervals between index and reference test were either moderately (33%-66%) or infrequently (<33%) reported. Studies in STARD-adopting journals (mean [SD], 20.5 [2.9] items in adopting journals vs 18.6 [2.3] items in nonadopting journals; *P* = .002) and studies citing STARD (mean [SD], 21.3 [0.9] items in citing studies vs 19.5 [2.9] items in nonciting studies; *P* = .01) reported more items. Variation by country and journal of publication were identified. No differences in STARD adherence were identified by body region imaged (mean [SD], abdominal, 20.0 [2.5] items; head and neck, 17.8 [1.6] items; musculoskeletal, 19.2 [3.1] items; thoracic, 20.2 [2.8] items; and other or procedural, 19.8 [2.7] items; *P* = .29), study design (mean [SD], prospective, 19.7 [2.9] items; retrospective, 19.7 [1.8] items; *P* > .99), patient population (mean [SD], pediatric, 20.0 [3.1] items; adult, 20.2 [2.7] items; mixed, 17.9 [1.9] items; *P* = .09), use of supplementary materials (mean [SD], yes, 19.2 [3.0] items; no, 19.7 [2.8] items; *P* = .91), or journal impact factor (mean [SD], higher impact factor, 20.3 [3.1] items; lower impact factor, 19.1 [2.4] items; *P* = .08).

**Conclusions and Relevance:**

Overall, the literature on acute care point-of-care ultrasound showed moderate adherence to the STARD 2015 guidelines, with more complete reporting found in studies citing STARD and those published in STARD-adopting journals. This study has established a current baseline for reporting; however, future studies are required to understand barriers to complete reporting and to develop strategies to mitigate them.

## Key Points

**Question:**

What is the completeness of reporting for the literature on acute point-of-care ultrasound, as indicated by adherence to the Standards for Reporting of Diagnostic Accuracy (STARD) 2015 guidelines?

**Findings:**

This systematic review of 74 studies found that overall adherence to STARD was moderate, with a mean of 19.7 of 30 items (66%) reported. Studies citing STARD and those published in journals endorsing STARD had a higher number of reported items.

**Meaning:**

These findings suggest that adherence of point-of-care ultrasound research to the STARD 2015 guidelines is moderate, which may limit the ability to detect bias in individual studies and prevent appropriate translation of research into clinical practice.

## Teaser

This systematic review assesses adherence to the Standards for Reporting of Diagnostic Accuracy (STARD) 2015 guidelines in the acute care point-of-care ultrasound literature.

## Introduction

Point-of-care ultrasound (POCUS) has become an important part of the diagnostic arsenal for the contemporary acute care physician.^1,2,3,4,5,6^ In contrast to consultative ultrasound, where a scan is performed by a technologist and then later interpreted by a radiologist, POCUS can diagnose abnormal physiology and pathology at the bedside. With the increasing availability of ultrasound machines in hospitals, clinics, and the prehospital setting, the number of clinicians using POCUS and the potential indications for its use continue to grow.^1,2,3,7,8,9^ The diagnostic accuracy of consultative ultrasound has been well studied for numerous applications^10,11,12,13,14^; however, the test characteristics of POCUS remain an area of active research.^6,9,15,16,17^

Studies of diagnostic accuracy can be of heterogeneous methodological quality and have variable completeness of reporting.^18^ Incomplete reporting can limit the ability to detect bias, determine generalizability of study results, and reproduce research. Ultimately, this leads to the inability to appropriately translate research into clinical practice. Incomplete reporting can also prevent informative and unbiased systematic reviews and meta-analyses from being performed.^19,20^ As the body of literature surrounding POCUS continues to grow, any deficiencies in reporting must be identified with the aim of implementing knowledge translation strategies to correct them.

In 2003, the Standards for Reporting of Diagnostic Accuracy Studies (STARD) group published a list of 25 essential items that should be reported in diagnostic accuracy research.^21^ The STARD group updated their reporting guideline in 2015 (hereafter referred to as STARD 2015), which now incorporates 30 essential items.^22^ These items have been deemed essential when interpreting primary diagnostic accuracy studies, and they allow readers to assess for bias and generalizability. To our knowledge, the current level of adherence to STARD 2015 is not known for the literature on acute care POCUS.

The objective of this study was to evaluate diagnostic accuracy studies published in the acute care medicine literature (emergency medicine, critical care, and anesthesia journals) for completeness of reporting, as defined by adherence to STARD 2015. This study will establish the current level of reporting and can serve as a call to action to improve completeness of reporting in deficient areas. As POCUS becomes further integrated into clinical practice, high-quality and completely reported research governing its use is essential.

## Methods

Research ethics board approval for this type of research is not required at the University of Ottawa because no human participants were involved. The search, data extraction, and data analyses were performed according to a prespecified protocol available on the Open Science Framework.^23^ This systematic review follows the Preferred Reporting Items for Systematic Reviews and Meta-analyses (PRISMA) reporting guideline.

### Data Sources

The search was performed on June 13, 2019, with assistance from an experienced medical research librarian. MEDLINE was searched for diagnostic accuracy studies evaluating POCUS published in critical care, emergency medicine, and anesthesia journals (as designated by Thompson Reuters Journal Citations Reports 2018).^24^ A date range of 2016 to 2019 was applied to evaluate articles published after the introduction of the updated STARD 2015 criteria. The search was performed using a previously published search filter for diagnostic accuracy studies.^25^ The full search strategy is available in eTable 1 in the Supplement.

### Study Selection

Studies were included if they met all of the following inclusion criteria: studies that examined the diagnostic accuracy of POCUS against a reference standard in human participants, studies that reported a measure of diagnostic accuracy (sensitivity, specificity, likelihood ratios, diagnostic odds ratio, or area under the receiver operating characteristic curve), and studies that were published in the English language. Point-of-care ultrasound was defined as ultrasound performed by nontechnologist, nonradiologist clinicians to distinguish it from consultative ultrasound. Studies were excluded if they evaluated predictive or prognostic tests or were reviews, meta-analyses, letters to the editor, or other commentaries.

Two reviewers (R.P. and J.B.) independently screened titles and abstracts to determine potential relevance. Any abstract that was deemed potentially relevant was automatically subject to full-text review. Full-text review was performed independently by 2 reviewers (R.P. and J.B.). Disagreements were resolved through consensus discussion with a third reviewer (T.A.M.).

### Data Extraction

Data were extracted independently by 2 reviewers (R.P., and one of J.B., H.K., or C.W.). Study characteristics extracted included study author, country of corresponding author’s institution, journal, journal impact factor in 2018, journal STARD endorsement included in the online instruction to authors (yes or no), year of publication, study design (prospective vs retrospective), patient population (pediatric vs adult vs mixed), use of supplementary material (yes or no), study citation of STARD (yes or no), and body region of POCUS scan (musculoskeletal vs head and neck vs thoracic vs abdominal vs skin and soft tissue vs procedural).

### Adherence to STARD 2015

Adherence to the STARD 2015 checklist was extracted independently and in duplicate (R.P., and one of J.B., H.K., or C.W.). When assessing adherence to the STARD 2015 checklist, each reporting requirement was rated as yes, no, or not applicable, with all disagreements resolved by consensus between the 2 reviewers. Items rated as not applicable were treated as a yes during data analysis. Several examples of how an item could potentially be not applicable are provided in eTable 2 in the Supplement. In addition, items with potentially unique aspects to diagnostic imaging and POCUS were divided into multiple subitems. This was based on a previous STARD 2015 checklist from Hong et al^26^ specific to diagnostic imaging, with POCUS-specific modifications made after a consensus discussion between 2 investigators (R.P. and T.A.M.).^26^ Items with multiple subpoints were scored with a total of 1 point per question, with fractional points awarded for each subitem (eg, 8.1 for setting, 8.2 for location, and 8.3 for dates were scored with 0.33 points per subitem). eTable 2 in the Supplement includes the STARD 2015 checklist with a detailed scoring rubric.

If an item was reported anywhere in the article, it was scored as a yes, unless STARD guidelines specified that it must be reported in a particular section (eg, item 1 in the title or abstract). Information included in either the full text report or the supplementary material (including online-only material) was scored as a yes. To optimize interobserver agreement, a training session was done for all reviewers using 2 articles. Interrater reliability was calculated and a κ value was provided.

### Statistical Analysis

The overall adherence to STARD 2015 was calculated for each item, subitem, and study. Yes and not applicable were scored as 1 point, and no was scored as 0 points. The maximum number of points for a study was 30. An arbitrary distinction of frequently reported (>66%), moderately reported (33%-66%), and infrequently reported (<33%) was used on the basis of a previously published scoring system.^26^

The Shapiro-Wilks test was used to confirm normal distribution. One-way analysis of variance was used to evaluate adherence to STARD by association with country, journal, body region, and patient population. A Tukey honest significant difference test was used for pairwise comparisons. The top 12 countries with the most included studies (because of a 3-way tie for tenth), the top 5 journals (most included studies), and 5 prespecified body regions were selected for evaluation. The 2-sided Welch *t* test was used to evaluate adherence to STARD on the basis of study design, STARD-adopting journals, use of supplemental materials, impact factor (median split), and STARD citation.

All data were stored in Excel spreadsheet software version 2013 (Microsoft Corp), and data analysis was performed using R statistical software version 3.1.2 (R Project for Statistical Computing). The level of statistical significance was set at *P* < .05 for all analyses. Data analysis was performed in November 2019.

## Results

### Search and Selection of Studies

The literature search yielded 399 unique results. One hundred six results were selected for full-text review, and 74 studies were included for analysis after full-text screening. Details of the study selection process and reasons for exclusion during full-text assessment are provided in the Figure. Characteristics of the included studies are summarized in Table 1. According to the country of the corresponding author, one-half of the studies were from the US (22 studies [30%]) and Turkey (14 studies [20%]). Most of the journals had adopted STARD (41 journals [55%]), and their median impact factor was 1.65 (range, 1.12-9.66). Most of the studies were prospective (68 studies [92%]) and most involved adult patients (44 studies [62%]).

**Figure.**
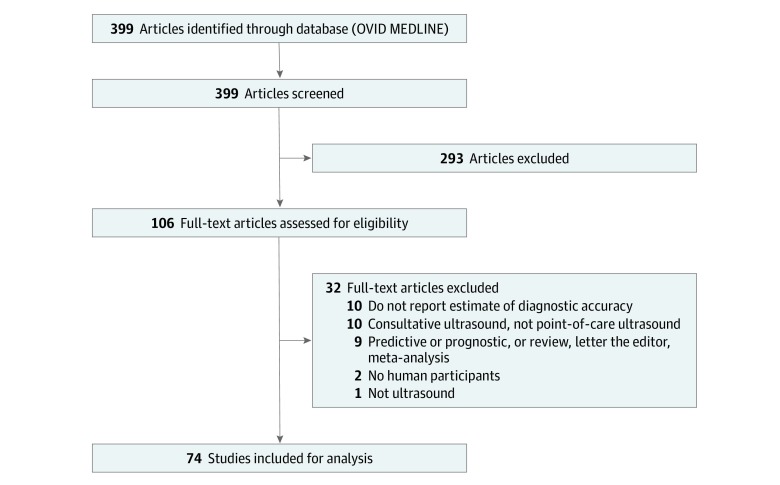
Study Flowchart

**Table 1.** Study Characteristics

Characteristics	Studies, No. (%) (N = 74)
Standards for Reporting of Diagnostic Accuracy items reported, mean (SD) (n = 30 items total)	19.7 (2.9)
Country of corresponding author	
US	22 (30)
Turkey	14 (20)
France	6 (8)
Canada	4 (5)
Australia	3 (4)
China	3 (4)
Italy	3 (4)
Spain	3 (4)
Others	16 (22)
Publishing in Standards for Reporting of Diagnostic Accuracy–adopting journals	
Yes	41 (55)
No	33 (45)
Journal of publication	
* American Journal of Emergency Medicine*	24 (32)
* Pediatric Emergency Care*	7 (9)
* The Journal of Emergency Medicine*	6 (8)
* Academic Emergency Medicine*	5 (7)
* Injury*	4 (5)
Other	28 (38)
Journal impact factor, median (range)	1.65 (1.12-9.66)
Body region of scan	
Thoracic	31 (42)
Abdominal	16 (22)
Musculoskeletal	16 (22)
Head and neck	6 (8)
Other or procedural	5 (7)
Study design	
Prospective	68 (92)
Retrospective	6 (8)
Patient population (n = 71)	
Adult	44 (62)
Pediatric	17 (24)
Mixed	10 (14)
Use of supplemental material	
Yes	8 (11)
No	66 (89)

### Adherence to STARD 2015

A summary of STARD 2015 adherence by item is presented in Table 2. Five of 74 studies cited STARD adherence in their methods. The mean (SD) number of STARD items reported for the 74 studies was 19.7 (2.9) of 30 items (66%), with a range from 13.8 to 25.8 items. The number of STARD items reported for each study is listed in eTable 3 in the Supplement. Interrater reliability was moderate (κ = 0.54).

**Table 2.** Reporting Frequency of Standards for Reporting of Diagnostic Accuracy 2015 Items^a^

Article section, item No.	Item description	Studies reporting the item, No. (%) (N = 74)
Title or abstract		
1	Identification as a study of diagnostic accuracy using at least 1 measure of accuracy (eg, sensitivity, specificity, predictive values, or area under the curve)	74 (100)
Abstract		
2	Structured summary of study design, methods, results, and conclusions	73 (99)
Introduction		
3	Scientific and clinical background, including the intended use and clinical role of the index test	74 (100)
4	Study objectives and hypotheses	74 (100)
Methods		
5	Whether data collection was planned before the index test and reference standard were performed (prospective study) or after (retrospective study)	72 (97)
6	Eligibility criteria	72 (97)
7	On what basis potentially eligible participants were identified (eg, symptoms, results from previous tests, and inclusion in registry)	72 (97)
8	Where and when potentially eligible participants were identified (setting, location, and dates)	
8.1	Setting	71 (96)
8.2^b^	Location	31 (42)
8.3	Dates	65 (89)
9^b^	Whether participants formed a consecutive, random, or convenience series	41 (55)
10	Index test, in sufficient detail to allow replication	
10.1	Details of imaging test provided in sufficient detail (multiple subitems)	
10.1a	Modality (transabdominal, transesophageal, transthoracic, or transbronchial)	74 (100)
10.1b	Vendor	65 (88)
10.1c	Model	60 (81)
10.1d	Technical parameters: probe type, transducer frequency, gray scale, Doppler	64 (86)
10.1e	Ultrasound contrast (if applicable)	74 (100)
10.2	Details of interpretation of the index test	
10.2a	No. of readers	56 (76)
10.2b	Level of training of readers	63 (85)
10.2c^b^	Images interpreted independently or in consensus	32 (43)
10.3	Reference standard, in sufficient detail to allow replication	71 (96)
11	Rationale for choosing the reference standard (if alternatives exist)	56 (76)
12		
12.1	Definition of and rationale for test positivity cutoffs or result categories of the index test, distinguishing prespecified from exploratory	
12.1a	Definition of test positivity cutoffs or result categories of the index test reported	63 (85)
12.1b^b^	Whether the test positivity cutoffs were prespecified vs exploratory	35 (47)
12.2	Definition of and rationale for test positivity cutoffs or result categories of reference standard, distinguishing prespecified from exploratory	
12.2a^b^	Definition of and rationale for test positivity cutoffs or result categories of the reference standard reported	46 (62)
12.2b^c^	Whether the test positivity cutoffs were prespecified vs exploratory	22 (30)
13		
13.1	Whether clinical information and reference standard results were available to the performers or readers of the index test	
13.1a^b^	Clinical information available to readers of the index test?	28 (38)
13.1b^b^	Reference standard results available to readers of the index test?	42 (57)
13.2	Whether clinical information and index test results were available to the assessors of the reference standard	
13.2a^b^	Clinical information available to assessors of the reference standard?	27 (36)
13.2b^b^	Index test results available to assessors of the reference standard?	41 (55)
14	Methods for estimating or comparing measures of diagnostic accuracy	74 (100)
15^c^	How indeterminate index test or reference standard results were handled	21 (28)
16^b^	How missing data on the index test and reference standard were handled	25 (34)
17	Any analyses of variability in diagnostic accuracy, distinguishing prespecified from exploratory	
17.1^b^	Analyses of variability	33 (45)
17.2^c^	Do they state which were prespecified vs exploratory?	7 (9)
18	Intended sample size and how it was determined	
18.1^b^	Intended sample size	25 (34)
18.2^c^	How sample size was determined	24 (32)
Results		
19^b^	Flow of participants, using a diagram	32 (43)
20	Baseline demographic and clinical characteristics of participants	65 (88)
21		
21.1	Distribution of severity of disease in those with the target condition	63 (85)
21.2^b^	Distribution of alternative diagnoses in those without the target condition	40 (54)
22	Time interval and any clinical interventions between the index test and the reference standard	
22.1^c^	Time interval	23 (31)
22.2^c^	Clinical interventions	19 (26)
23^b^	Cross-tabulation of the index test results (or their distribution) by the results of the reference standard	45 (61)
24	Did the study provide estimates of diagnostic accuracy and their precision?	68 (92)
25	Any adverse events from performing the index test or the reference standard	
25.1^c^	Index test	4 (5)
25.2^c^	Reference standard	9 (12)
Discussion		
26	Study limitations, including sources of potential bias, statistical uncertainty, and generalizability	
26.1	Sources of potential bias	70 (95)
26.2^b^	Potential sources of statistical uncertainty reported?	39 (53)
26.3	Generalizability	61 (82)
27	Implications for practice, including the intended use and clinical role of the index test	71 (96)
Other information		
28^c^	Registration No. and name of registry	9 (12)
29^c^	Where the full study protocol can be accessed	9 (12)
30	Sources of funding and other support; role of funders	
30.1	Sources of funding and other support	54 (73)
30.2	Role of funders	52 (70)

^a^ Frequently reported studies (>66%) do not have a footnote.

^b^ Moderately reported items (33%-66% of studies).

^c^ Infrequently reported items (<33% of studies).

Twenty-eight of the 30 items were frequently reported in whole, or in part (subitems), characterized by a reporting frequency of greater than 66%. Of note, the total number of frequently, moderately, and infrequently reported items is greater than 30 because some subitems are present in different categories. Some of the frequently reported items are of particular relevance to POCUS, including item 10.1 (a full description of the modality, equipment, and parameters of the ultrasound machine; reported by 74 studies [100%], 60 studies [81%], and 64 studies [86%], respectively), subitem 10.2b (the level of training of readers; reported by 63 studies [85%]), and subitem 10.3 (a clear description of the reference standard in sufficient detail to allow replication; reported by 71 studies [96%]).

Sixteen of the 30 items were moderately reported, in whole or in part (subitems), characterized by a reporting frequency of 33% to 66% (Table 2). Several items are particularly relevant to POCUS and are essential when assessing risk of bias. These include item 9 (whether participants formed a consecutive, convenience, or random sample; reported by 41 studies [55%]), and item 10.2c (whether images were interpreted independently or in consensus; reported by 32 studies [43%]). Notably, all subitems of item 13 were only moderately reported (whether readers of the index and reference tests were blinded to clinical data, and to each other).

Ten of the 30 items were infrequently reported, in whole or in part (subitems), characterized by a reporting frequency of less than 33% (Table 2). Some of these items are particularly relevant to POCUS and are essential when assessing risk of bias. These include item 15 (how indeterminate tests were handled; reported by 21 studies [28%]), subitem 17.2 (whether analyses of subgroups and heterogeneity were prespecified or exploratory; reported by 7 studies [9%]), and subitems 22.1 (the time interval between the index and reference test; reported by 23 studies [31%]) and 22.2 (whether any clinical interventions were performed between the index and reference test; reported by 19 studies [26%]).

### Subgroup Analyses

Subgroup analyses of prespecified variables were performed and are summarized in Table 3. Additional details of the subgroup analyses are provided in eTables 4, 5, 6, 7, 8, 9, 10, 11, and 12 in the Supplement. The Shapiro-Wilks test confirmed the data are normally distributed (*P* = .41).

**Table 3.** Summary of Subgroup Analysis

Subgroup	Summary of finding	STARD items, mean (SD), No.	*P* value
Country of corresponding author	Higher No. of STARD items when France was compared with Turkey	22.1 (2.4) vs 17.6 (1.9)	.04^a^
STARD-adopting journal	Higher No. of items reported in STARD-adopting journals compared with nonadopting journals	20.5 (2.9) vs 18.6 (2.3)	.002^b^
Citation of STARD in article	Higher No. of items reported in STARD citing studies compared with nonciting studies	21.3 (0.9) vs 19.5 (2.9)	.01^b^
Journal of publication	Higher No. of STARD items in *Academic Emergency Medicine* and *The Journal of Emergency Medicine* compared with the *American Journal of Emergency Medicine*	21.1 (2.2) vs 18.1 (2.1)	.002^a^
		22.0 (1.9) vs 18.1 (2.1)	.02^a^
Journal impact factor (median split)	No statistically significant difference between studies in higher impact factor compared with lower impact factor journals	20.3 (3.1) vs 19.1 (2.4)	.08^b^
Supplementary material	No statistically significant difference between studies with supplemental materials compared with those without supplemental materials	19.2 (3.0) vs 19.7 (2.8)	.91^b^
Patient population	No statistically significant difference between pediatric, adult, and mixed population studies	20.0 (3.1) vs 20.2 (2.7) vs 17.9 (1.9)	. 09^c^
Study design	No statistically significant difference between prospective or retrospective studies	19.7 (2.9) vs 19.7 (1.8)	>.99^b^
Body region	No statistically significant difference between body regions scanned (abdominal, head and neck, musculoskeletal, thoracic, and other or procedural)	20.0 (2.5) vs 17.8 (1.6) vs 19.2 (3.1) vs 20.2 (2.8) vs 19.8 (2.7)	.29^c^

Abbreviation: STARD, Standards for Reporting of Diagnostic Accuracy.

^a^ Analysis of variance with Tukey honest significant difference test.

^b^ Two-tailed *t* test.

^c^ Analysis of variance.

Studies published in STARD-adopting journals had a higher number of reported items compared with nonadopting journals (mean [SD], 20.5 [2.9] items vs 18.6 [2.3] items; *P* = .002). Studies that cited STARD had a higher number of reported items compared with nonciting studies (mean [SD], 21.3 [0.9] items vs 19.5 [2.9] items; *P* = .01). Variation by country and journal of publication were identified. A Tukey honestly significant difference test showed a difference based on country of corresponding author when France was compared with Turkey (mean [SD], 22.1 [2.4] items vs 17.6 [1.9] items; *P* = .04). In addition, studies published in *Academic Emergency Medicine* and *The Journal of Emergency Medicine* had a statistically significantly higher number of reported items compared with the *American Journal of Emergency Medicine* (mean [SD], 21.1 [2.2] items and 22.0 [1.9] items vs 18.1 [2.1] items; *P* = .002 and *P* = .02, respectively). There was no difference in the number of STARD items reported according to body region scanned (mean [SD], abdominal, 20.0 [2.5] items; head and neck, 17.8 [1.6] items; musculoskeletal, 19.2 [3.1] items; thoracic, 20.2 [2.8] items; and other or procedural, 19.8 [2.7] items; *P* = .29), study design (mean [SD], prospective, 19.7 [2.9] items; retrospective, 19.7 [1.8] items; *P* > .99), patient population (mean [SD], pediatric, 20.0 [3.1] items; adult, 20.2 [2.7] items; mixed, 17.9 [1.9] items; *P* = .09), use of supplementary materials (mean [SD], yes, 19.2 [3.0] items; no, 19.7 [2.8] items; *P* = .91), or journal impact factor (mean [SD], higher impact factor, 20.3 [3.1] items; lower impact factor, 19.1 [2.4] items; *P* = .08).

## Discussion

The completeness of reporting of the acute care POCUS literature, defined as adherence to STARD 2015, was moderate with a mean (SD) of 19.7 (2.9) of 30 items (66%) being reported. The STARD reporting varied according to country of corresponding author, citation of STARD in the article, journal of publication, and whether the journal of publication endorsed STARD in the instructions to authors. Reporting did not vary on the basis of impact factor, study design, patient population, use of supplemental materials, or body region.

Items pertaining to the technical parameters of ultrasound (ie, machine model, details of scan, and probe specifications) and to the readers of POCUS were frequently reported; these are essential items to consider when evaluating the applicability of a study to clinical practice. For example, image quality can vary with machine make and model, which could limit reproducibility and generalizability of study results depending on equipment availability in a certain clinical setting. Point-of-care ultrasound is also highly operator dependent, and its accuracy varies with practitioner expertise.^27,28^ This makes it important to report operator expertise and any specific training received to learn a scan (eg, workshops) to allow other clinicians to assess the feasibility of integrating a new ultrasound scan into their own practice.

Although many items were frequently reported, the image interpretation practices (individual vs consensus reading), blinding to the reference standard and clinical information, and analysis of heterogeneity in the data were only moderately or infrequently reported (Table 2). Deficiencies in these areas of reporting are troublesome, because they can easily lead to bias and limit translation of research into clinical practice. Lack of blinding of the index test to the reference standard and failure to specify whether subgroup analyses are prespecified have both been shown to cause bias in diagnostic accuracy research and are included in the currently recommended risk of bias tool for assessing diagnostic accuracy studies.^18^

The observed deficiencies in reporting are not unique to this study and are similar to previous analyses of the diagnostic imaging literature.^26,29^ Hong et al^26^ investigated adherence to STARD 2015 for multiple imaging modalities. They found a lower number of STARD items reported compared with our sample (mean [SD],16.6 [2.21] of 30 items [55%]),^26^ and similar deficiencies in reporting on a per-item basis. In their subgroup of consultative ultrasound studies, the mean (SD) STARD adherence was 16.7 (2.05) of 30 items (55%)^26^; however, given potential confounders with study design and sample size, a direct comparison would be at high risk of bias. This suggests that any deficiencies in reporting may not be unique to POCUS but are more indicative of a global deficiency in the reporting of diagnostic imaging studies. A recent study by Thiessen et al^29^ assessed adherence of POCUS studies to the original STARD criteria (published in 2003) in 5 emergency medicine journals from 2005 to 2010. They found a mean of 15 of 25 (60%) STARD items reported.^29^ Several key differences in methods, including different scoring rubrics and their inclusion of studies not reporting diagnostic accuracy, limits direct comparison with our sample.

In the present study, blinding of the POCUS reader to clinical data was only moderately reported. Point-of-care ultrasound is performed and interpreted by clinicians at the bedside, making clinical information an important potential source of bias. For example, if the history and physical examination are suggestive of a fracture, a clinician performing POCUS may search with the ultrasound until a fracture is identified. This highlights a distinction between POCUS practice and research. In practice, POCUS is often thought of as an extension of the physical examination. During POCUS research, however, blinding to clinical information should be clearly reported. This helps readers evaluate the generalizability of the results and assess for inadvertent inclusion of clinical history and physical examination maneuvers in the POCUS accuracy estimates.

Several other infrequently reported STARD items include the time elapsed and any clinical interventions performed between the index test and reference standard. Point-of-care ultrasound is often used to diagnose acute and dynamic conditions (eg, heart failure or elevated intracranial pressure) that have the potential to rapidly improve or progress either spontaneously or through interventions. Delay in performing the reference standard has the potential to introduce false-positive or false-negative findings depending on the course of the acute illness. Certain procedures (eg, chest tube insertion for pneumothorax) also have the potential to entirely reverse the pathology identified by POCUS, potentially creating incorrect false-positive results.

Another notable finding was that there was a higher number of items reported in journals that endorse STARD in their instructions to authors; this is similar to previous evaluations and may be associated with STARD-adopting journals using the STARD 2015 checklist in their peer review process, or authors being prompted to adhere to STARD through the online instructions to authors.^26^ There was also a higher number of items reported in the 5 of 74 studies that cited STARD adherence in their methods. Adherence to reporting guidelines should be of interest to authors and journal editors alike, because it may be associated with higher citation rates; however, the literature^30^ is conflicting with a study by Dilauro et al^31^ showing that the association of STARD adherence with citation rate did not persist after controlling for journal impact factor. Despite this, only a small minority of the studies cited STARD adherence in their methods, suggesting either a lack of awareness regarding the STARD 2015 guidelines, lack of enforcement of reporting guidelines by journals, or other barriers to adherence.

### Limitations

Our literature search was only applied to journals listed in the categories of critical care, emergency medicine, and anesthesia as defined by the Thompson Reuters Journal Citations Reports 2018, and, therefore, our results may not be generalizable to POCUS research in other clinical settings. Additionally, although the study identified deficiencies in reporting, reasons for incomplete reporting were not assessed. Furthermore, because subgroups were prespecified, some categories have a small number of studies and post hoc recategorization was not performed to avoid introducing bias. Considering this, the study may have been underpowered to detect a difference in STARD adherence in some subgroups, including by journal impact factor (*P* = .08), which has previously been shown to vary between studies published in high–impact factor and low–impact factor journals.^26^ Furthermore, although a statistically significant difference between STARD-adopting journals compared with nonadopting journals was found, it is unclear how clinically important such a small difference would be to the reader of a study, because some STARD items have the potential to introduce more bias compared with others.

## Conclusions

The role of POCUS in the diagnosis and management of acutely ill patients is continuing to expand. The ability to integrate POCUS into clinical practice relies on accurate estimates for the diagnostic accuracy of each scan. In this study, adherence of POCUS research to STARD 2015 was only moderate, which may limit the ability to detect bias in individual studies and prevent appropriate translation of research into clinical practice.

## Notes

Supplement. **eTable 1.** Full MEDLINE Search Strategy **eTable 2.** Modified STARD 2015 Checklist for Data Extraction **eTable 3.** Complete List of Included Studies With STARD Adherence **eTable 4.** Subgroup Analysis of STARD Adherence by Study Design **eTable 5.** Subgroup Analysis of STARD Adherence by Country **eTable 6.** Subgroup Analysis of STARD Adherence by Body System **eTable 7.** Subgroup Analysis by STARD Adoption by Journal **eTable 8.** Subgroup Analysis by STARD Citation in Article **eTable 9.** Subgroup Analysis by Patient Population (Adult vs Pediatric vs Mixed) **eTable 10.** Subgroup Analysis of the Five Most Common Journals **eTable 11.** Subgroup Analysis by Use of Supplemental Materials **eTable 12.** Subgroup Analysis by Impact Factor (Median Split)

## References

[1] Arntfield RT, Millington SJ Point of care cardiac ultrasound applications in the emergency department and intensive care unit: a review. Curr Cardiol Rev. 2012;8(2):98-108. doi:10.2174/157340312801784952 22894759 PMC3406278

[2] Whitson MR, Mayo PH Ultrasonography in the emergency department. Crit Care. 2016;20(1):227. doi:10.1186/s13054-016-1399-x 27523885 PMC4983783

[3] Sippel S, Muruganandan K, Levine A, Shah S Review article: use of ultrasound in the developing world. Int J Emerg Med. 2011;4:72. doi:10.1186/1865-1380-4-72 22152055 PMC3285529

[4] Andersen CA, Holden S, Vela J, Rathleff MS, Jensen MB Point-of-care ultrasound in general practice: a systematic review. Ann Fam Med. 2019;17(1):61-69. doi:10.1370/afm.2330 30670398 PMC6342599

[5] de Groot-de Laat LE, ten Cate FJ, Vourvouri EC, van Domburg RT, Roelandt JR Impact of hand-carried cardiac ultrasound on diagnosis and management during cardiac consultation rounds. Eur J Echocardiogr. 2005;6(3):196-201. doi:10.1016/j.euje.2004.09.013 15894238

[6] Kim DJ, Francispragasam M, Docherty G, Test characteristics of point-of-care ultrasound for the diagnosis of retinal detachment in the emergency department. Acad Emerg Med. 2019;26(1):16-22. 29774966 10.1111/acem.13454

[7] Prager R, Sedgwick C, Lund A, Prospective evaluation of point-of-care ultrasound at a remote, multi-day music festival. Prehosp Disaster Med. 2018;33(5):484-489. doi:10.1017/S1049023X18000821 30269693

[8] Marbach JA, Almufleh A, Di Santo P, Comparative accuracy of focused cardiac ultrasonography and clinical examination for left ventricular dysfunction and valvular heart disease: a systematic review and meta-analysis. Ann Intern Med. Published online August 6, 2019. doi:10.7326/M19-1337 31382273

[9] Maw AM, Hassanin A, Ho PM, Diagnostic accuracy of point-of-care lung ultrasonography and chest radiography in adults with symptoms suggestive of acute decompensated heart failure: a systematic review and meta-analysis. JAMA Netw Open. 2019;2(3):e190703. doi:10.1001/jamanetworkopen.2019.0703 30874784 PMC6484641

[10] Remonti LR, Kramer CK, Leitão CB, Pinto LC, Gross JL Thyroid ultrasound features and risk of carcinoma: a systematic review and meta-analysis of observational studies. Thyroid. 2015;25(5):538-550. doi:10.1089/thy.2014.0353 25747526 PMC4447137

[11] Giljaca V, Nadarevic T, Poropat G, Nadarevic VS, Stimac D Diagnostic accuracy of abdominal ultrasound for diagnosis of acute appendicitis: systematic review and meta-analysis. World J Surg. 2017;41(3):693-700. doi:10.1007/s00268-016-3792-7 27864617

[12] Wang C, Yu C, Yang F, Yang G Diagnostic accuracy of contrast-enhanced ultrasound for renal cell carcinoma: a meta-analysis. Tumour Biol. 2014;35(7):6343-6350. doi:10.1007/s13277-014-1815-2 24659450

[13] Wertz JR, Lopez JM, Olson D, Thompson WM Comparing the diagnostic accuracy of ultrasound and CT in evaluating acute cholecystitis. AJR Am J Roentgenol. 2018;211(2):W92-W97. doi:10.2214/AJR.17.18884 29702020 PMC6082629

[14] Richardson A, Gallos I, Dobson S, Campbell BK, Coomarasamy A, Raine-Fenning N Accuracy of first-trimester ultrasound in diagnosis of tubal ectopic pregnancy in the absence of an obvious extrauterine embryo: systematic review and meta-analysis. Ultrasound Obstet Gynecol. 2016;47(1):28-37. doi:10.1002/uog.14844 25766776

[15] Wong C, Teitge B, Ross M, Young P, Robertson HL, Lang E The accuracy and prognostic value of point-of-care ultrasound for nephrolithiasis in the emergency department: a systematic review and meta-analysis. Acad Emerg Med. 2018;25(6):684-698. doi:10.1111/acem.13388 29427476

[16] Parker BK, Salerno A, Euerle BD The use of transesophageal echocardiography during cardiac arrest resuscitation: a literature review. J Ultrasound Med. 2019;38(5):1141-1151. doi:10.1002/jum.14794 30280396

[17] Lahham S, Shniter I, Thompson M, Point-of-care ultrasonography in the diagnosis of retinal detachment, vitreous hemorrhage, and vitreous detachment in the emergency department. JAMA Netw Open. 2019;2(4):e192162. doi:10.1001/jamanetworkopen.2019.2162 30977855 PMC6481597

[18] Whiting PF, Rutjes AW, Westwood ME, ; QUADAS-2 Group QUADAS-2: a revised tool for the quality assessment of diagnostic accuracy studies. Ann Intern Med. 2011;155(8):529-536. doi:10.7326/0003-4819-155-8-201110180-00009 22007046

[19] Tunis AS, McInnes MD, Hanna R, Esmail K Association of study quality with completeness of reporting: have completeness of reporting and quality of systematic reviews and meta-analyses in major radiology journals changed since publication of the PRISMA statement? Radiology. 2013;269(2):413-426. doi:10.1148/radiol.13130273 23824992

[20] Frank RA, Bossuyt PM, McInnes MDF Systematic reviews and meta-analyses of diagnostic test accuracy: the PRISMA-DTA statement. Radiology. 2018;289(2):313-314. doi:10.1148/radiol.2018180850 30015590

[21] Bossuyt PM, Reitsma JB, Bruns DE, ; Standards for Reporting of Diagnostic Accuracy Towards complete and accurate reporting of studies of diagnostic accuracy: the STARD Initiative. Radiology. 2003;226(1):24-28. doi:10.1148/radiol.2261021292 12511664

[22] Bossuyt PM, Reitsma JB, Bruns DE, ; STARD Group STARD 2015: an updated list of essential items for reporting diagnostic accuracy studies. Radiology. 2015;277(3):826-832. doi:10.1148/radiol.2015151516 26509226

[23] OSF Registries Acute care POCUS: adherence to STARD 2015. Published November 24, 2019. Accessed November 31, 2019. https://osf.io/2h8s9

[24] Thomson Reuters Incites journal citation reports. Published 2018 Accessed June 15, 2019. https://incites.clarivate.com

[25] Devillé WL, Bezemer PD, Bouter LM Publications on diagnostic test evaluation in family medicine journals: an optimal search strategy. J Clin Epidemiol. 2000;53(1):65-69. doi:10.1016/S0895-4356(99)00144-4 10693905

[26] Hong PJ, Korevaar DA, McGrath TA, Reporting of imaging diagnostic accuracy studies with focus on MRI subgroup: adherence to STARD 2015. J Magn Reson Imaging. 2018;47(2):523-544. doi:10.1002/jmri.25797 28640484

[27] Kim J, Kim K, Kim J, The learning curve in diagnosing acute appendicitis with emergency sonography among novice emergency medicine residents. J Clin Ultrasound. 2018;46(5):305-310. doi:10.1002/jcu.22577 29315613

[28] Tsou PY, Chen KP, Wang YH, Diagnostic accuracy of lung ultrasound performed by novice versus advanced sonographers for pneumonia in children: a systematic review and meta-analysis. Acad Emerg Med. 2019;26(9):1074-1088. doi:10.1111/acem.13818 31211896

[29] Thiessen M, Vogel JA, Byyny RL, Emergency ultrasound literature and adherence to standards for reporting of diagnostic accuracy criteria. J Emerg Med. Published online November 7, 2019. doi:10.1016/j.jemermed.2019.09.029 31708317 PMC7202948

[30] van der Pol CB, McInnes MD, Petrcich W, Tunis AS, Hanna R Is quality and completeness of reporting of systematic reviews and meta-analyses published in high impact radiology journals associated with citation rates? PLoS One. 2015;10(3):e0119892. doi:10.1371/journal.pone.0119892 25775455 PMC4361663

[31] Dilauro M, McInnes MD, Korevaar DA, Is there an association between STARD statement adherence and citation rate? Radiology. 2016;280(1):62-67. doi:10.1148/radiol.2016151384 26836050

