# Supplementary material for: Adherence to the Standards for Reporting of Diagnostic Accuracy (STARD) 2015 Guidelines in Acute Point-of-Care Ultrasound Research
Source: JAMA Netw Open. 2020 May 1;3(5):e203871. doi: 10.1001/jamanetworkopen.2020.3871 (PMC7195624; doi:10.1001/jamanetworkopen.2020.3871)
Supplement: Supplement. — eTable 1. Full MEDLINE Search Strategy eTable 2. Modified STARD 2015 Checklist for Data Extraction eTable 3. Complete List of Included Studies With STARD Adherence eTable 4. Subgroup Analysis of STARD Adherence by Study Design eTable 5. Subgroup Analysis of STARD Adherence by Country eTable 6. Subgroup Analysis of STARD Adherence by Body System eTable 7. Subgroup Analysis by STARD Adoption by Journal eTable 8. Subgroup Analysis by STARD Citation in Article eTable 9. Subgroup Analysis by Patient Population (Adult vs Pediatric vs Mixed) eTable 10. Subgroup Analysis of the Five Most Common Journals eTable 11. Subgroup Analysis by Use of Supplemental Materials eTable 12. Subgroup Analysis by Impact Factor (Median Split) [file jamanetwopen-3-e203871-s001.pdf]

Supplementary Online Content

Prager R, Bowdridge J, Kareemi H, Wright C, McGrath TA, McInnes MDF. Adherence to the Standards for Reporting of Diagnostic Accuracy (STARD) 2015 guidelines in acute point-of-care ultrasound research. *JAMA Netw Open*. 2020;3(5):e203871. doi:10.1001/jamanetworkopen.2020.3871

eTable 1. Full MEDLINE Search Strategy

eTable 2. Modified STARD 2015 Checklist for Data Extraction

eTable 3. Complete List of Included Studies With STARD Adherence

eTable 4. Subgroup Analysis of STARD Adherence by Study Design

eTable 5. Subgroup Analysis of STARD Adherence by Country

eTable 6. Subgroup Analysis of STARD Adherence by Body System

eTable 7. Subgroup Analysis by STARD Adoption by Journal

eTable 8. Subgroup Analysis by STARD Citation in Article

eTable 9. Subgroup Analysis by Patient Population (Adult vs Pediatric vs Mixed)

eTable 10. Subgroup Analysis of the Five Most Common Journals

eTable 11. Subgroup Analysis by Use of Supplemental Materials

eTable 12. Subgroup Analysis by Impact Factor (Median Split)

This supplementary material has been provided by the authors to give readers additional information about their work.

© 2020 Prager R et al. *JAMA Network Open.*

eTable 1. Full MEDLINE Search Strategy

	Search Term
1	"Sensitivity and Specificity"
2	sensitivit*.mp.
3	specificit*.mp.
4	false negative.tw.
5	accuracy.tw.
6	1 or 2 or 3 or 4 or 5
7	ultraso*.mp. (limit yr 2016 – 2019)
8	POCUS.mp. (limit yr 2016 – 2019)
9	echo*.mp. (limit yr 2016-2019)
10	"Point of Care".mp. (limit yr 2016-2019)
11	sono*.mp (limit yr 2016-2019)
12	7 or 8 or 9 or 10 or 11 (limited to English and Human Subjects)
13	Search by Journal (Lancet Respiratory Medicine or Intensive Care Medicine or American Journal of Respiratory and Critical Care Medicine or CHEST or Critical Care Medicine or Critical Care London England or Resuscitation or Journal of Neurotrauma or Annals of Intensive Care or Journal of Trauma and Acute Care Surgery or Seminars in Respiratory and Critical Care Medicine or Neurocritical Care or Current Opinion in Critical Care or Pediatric Critical Care Medicine or Shock or Journal of Critical Care or Minerva Anestesiologica or Journal of Intensive Care Medicine or Human Gene Therapy or Critical Care Clinics or Burns or Respiratory Care or American Journal of Critical Care or Therapeutic Hypothermia and Temperature Management or Critical Care and Resuscitation or Australian Critical Care or Medicina Intensiva or Critical Care Nurse or anasthesie intensivtherapie notfallmedizin or anasthesiologie intensivmedizin notfallmedizin schmerztherapie or Journal of Trauma Nursing or Anaesthesia and Intensive Care or Annals of Emergency Medicine or Emergencias or World Journal of Emergency Surgery or Academic Emergency Medicine or Scandinavian Journal of Trauma Resuscitation and Emergency Medicine or Prehospital Emergency Care or Emergency Medicine Journal or European Journal of Emergency Medicine or European Journal of Trauma and Emergency Surgery or Canadian Journal of Emergency Medical Care or Emergency Medicine Clinics of North America or Emergency Medicine Australasia or American Journal of Emergency Medicine or Journal of Emergency Medicine or Pediatric Emergency Care or Prehospital and Disaster Medicine or Unfallchirug or Journal of Emergency Nursing or Notfall & rettungsmedizin or ulusal travma ve acil cerrahi dergisi turkish journal of trauma & emergency surgery tjtes or emergency medicine international print or anesthesiology or british journal of anaesthesia or pain or anaesthesia or regional anesthesia & pain medicine or European Journal of Anaesthesiology or Anesthesia and Analgesia or International Journal of Obstretic Anesthesia or Canadian Journal of Anaesthesia or Journal of Neurosurgical Anesthesiology or Clinical Journal of Pain or European Journal of Pain or Pain Medicine or Current Opinion in Anaesthesiology or Pain Physician or Journal of Clinical Monitoring and Computing or Acta anaesthesiologica or Anaesthesia Critical Care & Pain Medicine or Pain Practice or Journal of Clinical Anesthesia or BMC Anesthesiology or Journal of Cardiothoracic and Vascular Anesthesia or Journal of Anesthesia or Anaesthesist or revista brasileira de anestesiologia or paediatric aneathesia or der schmerz
14	6 and 12 and 13

© 2020 Prager R et al. *JAMA Network Open.*

eTable 2: Modified STARD 2015 Checklist for Data Extraction

Section & Topic	No.		Item
**TITLE OR ABSTRACT**			
	1		Identification as a study of diagnostic accuracy using at least one measure of accuracy (such as sensitivity, specificity, predictive values, or AUC)<br>Note: Must state “diagnostic performance/ accuracy”. Only “diagnosis” or “accuracy” or “performance” alone are not sufficient.
**ABSTRACT**			
	2		Structured summary of study design, methods, results, and conclusions<br>Note: Any summary with sub-headings is sufficient.
**INTRODUCTION**			
	3		Scientific & clinical background, including the intended use and clinical role of the index test<br>Note: At minimum, the study should mention alternative diagnostic tests in the clinical pathway and the rationale for performing the study.
	4		Study objectives and hypotheses
**METHODS**			
Study design	5		Whether data collection was planned before the index test and reference standard were performed (prospective study) or after (retrospective study)<br>Note: Sufficient if the study explicitly states “prospective” or “retrospective” design.
Participants	6		Eligibility criteria<br>Note: At minimum, the study must have explicitly stated inclusion and exclusion criteria.
	7		On what basis potentially eligible participants were identified (such as symptoms, results from previous tests, inclusion in registry)<br>Note: Examples include, but are not limited to, presenting symptoms and referral by a healthcare professional.
	8		Where and when potentially eligible participants were identified (setting, location and dates).
		8.1	Setting<br>Note: Need to explicitly state department or clinical unit where patient recruitment took place. Corresponding author information is not considered sufficient.
		8.2	Location<br>Note: At least city/ hospital must be reported. Corresponding author information is not considered sufficient. (i.e. “our medical center” is not acceptable)
		8.3	Dates<br>Note: Start and end date must be reported; year (not month) is the minimum requirement.
	9		Whether participants formed a consecutive, random or convenience series<br>Note: Must explicitly state how patients were enrolled and use specific terms- consecutive, random or convenience (or equivalent synonyms).
Test methods	10		Index test, in sufficient detail to allow replication
		10-1	Details of imaging test provided in sufficient detail (multiple sub-items)<br>a) Modality (transthoracic, transabdominal, transesophageal, transbronchial, etc.)<br>b) Vendor<br>c) Model<br>d) Technical parameters: probe type, transducer frequency, grey scale, Doppler, etc<br>e) Ultrasound contrast if used
		10-2	Details of interpretation of the index test (multiple sub-items)<br>a) Number of readers<br>b) Level of training of readers

© 2020 Prager R et al. *JAMA Network Open.*

			c) Images interpreted independently or in consensus (if only one reviewer then this should be answered as 'N/A')
		10-3	Reference standard, in sufficient detail to allow replication<br>Note: At minimum, the study needs to have a description of the reference standard. Examples include (but are not limited to):<br>1) Diagnostic imaging – description of the diagnostic imaging performed<br>2) Surgery – description of the type of surgery performed<br>3) Histology/pathology – description of the type of staining performed<br>4) Follow-up – description of modality used and duration of follow-up
	11		Rationale for choosing the reference standard (if alternatives exist)<br>Note: Acceptable if the study explains anywhere in the paper (even discussion). Simply indicating that alternatives exist is not sufficient.
	12.1		Definition of and rationale for test positivity cut-offs or result categories of the index test, distinguishing pre-specified from exploratory
		12.1.a	Definition of test positivity cut-offs or result categories of the index test reported
		12.1.b	Do they state pre-specified vs. exploratory? Note: Must explicitly state one vs. the other, not inferring; if a = 'no' then report b as 'no'.
	12.2		Definition of and rationale for test positivity cut-offs or result categories of reference standard, distinguishing pre-specified from exploratory
		12.2.a	Definition of and rationale for test positivity cut-offs or result categories of the reference standard reported<br>Note: Needs to be explicit even if it could be implicitly concluded that the same criteria were used as that of the index test
		12.2.b	Do they state pre-specified vs. exploratory? Note: Must explicitly state one vs. the other, not inferring; if a = 'no' then report b as 'no'.
	13.1		Whether clinical information and reference standard results were available to the performers/readers of the index test.
		13.1.a	Clinical information available to readers of the index test?
		13.2.b	Reference standard results available to readers of the index test?<br>Note: Need to explicitly state whether the reference standard results were available even if it could be implicitly concluded based on the implied order of testing.
	13.2		Whether clinical information and index test results were available to the assessors of the reference standard
		13.2.a	Clinical information available to assessors of the reference standard?
		13.2.b	Index test results available to assessors of the reference standard?
Analysis	14		Methods for estimating or comparing measures of diagnostic accuracy (i.e. do they state how they will calculate or compare diagnostic accuracy measures?)
	15		How indeterminate index test or reference standard results were handled
	16		How missing data on the index test and reference standard were handled<br>Note: Sufficient if they report something about missing data, regardless of to which test (index test or reference standard) it applies.
	17		Any analyses of variability in diagnostic accuracy, distinguishing pre-specified from exploratory
		17.1	Analyses of variability.<br>Note: any subgroup analysis(es) or presentation of data for separate readers would be considered sufficient.
		17.2	Do they state which were pre-specified vs. exploratory? Note: if 17.1 is no, this should be 'no'.<br>Note: It is considered pre-specified if the authors previously published a protocol on this subject or if the study mentions "pre-specified".
	18		Intended sample size and how it was determined

© 2020 Prager R et al. *JAMA Network Open.*

		18.1	Intended sample size
		18.2	How sample size was determined<br>Note: if 18.1 is no, this should be 'no'.
**RESULTS**			
Participants	19		Flow of participants, using a diagram
	20		Baseline demographic and clinical characteristics of participants<br>Note: must report at least age and gender (mean or median of each)
	21.1		Distribution of severity of disease in those with the target condition<br>Note: they must report more than just 2x2 data if there were more than yes/no for disease classification e.g. if there are 5 grades of disease and they consider positive >2 they must report the distribution of those with ratings of 1-5 not just those >2 and those not
	21.2		Distribution of alternative diagnoses in those without the target condition
	22		Time interval and any clinical interventions between the index test and the reference standard
		22.1	Time interval<br>Note: It will be considered sufficient if they report 'all ref tests done within x-time of reference standard'
		22.2	Clinical interventions<br>Note: Any mention of any clinical interventions will be considered "yes." The clinical intervention must be performed between index test and the reference standard.
Test results	23		Cross tabulation of the index test results (or their distribution) by the results of the reference standard<br>Note: Sufficient if the study reports this "in text", as long if the exact numbers of the 2x2 tables were provided. E.g. "sensitivity was 90% (90/100).
	24		Did the study provide estimates of diagnostic accuracy and their precision? Note: Must report both point estimates and some measure of precision (95% CI, standard deviation or any measure of variability about the mean is considered acceptable).
	25		Any adverse events from performing the index test or the reference standard
		25.1	Index test
		25.2	Reference standard
**DISCUSSION**			
	26		Study limitations, including sources of potential bias, statistical uncertainty, and generalizability
		26.1	Sources of potential bias<br>Note: Any mention of any source of bias is sufficient
		26.2	Potential sources of statistical uncertainty reported? (i.e. discussion of impact of wide confidence intervals, sample size or statistical power)<br>Note: Any mention of any source of statistical uncertainty is acceptable and there is no need for discussion of these sources
		26.3	Generalizability<br>Note: Needs to indicate whether the study is applicable to other sub-groups or population.
	27		Implications for practice, including the intended use and clinical role of the index test
**OTHER INFORMATION**			
	28		Registration number and name of registry (if not registered, answer as 'no')
	29		Where the full study protocol can be accessed<br>Note: Considered sufficient if they state "full protocol can be obtained from the corresponding author" or if trial registered in an online registry
	30		Sources of funding and other support; role of funders
		30.1	Sources of funding and other support<br>Note: Statement of conflicts of interest is considered 'yes'
		30.2	Role of funders<br>Note: Rate as 'NA' if 30.1 is 'Yes' but no funding is declared, rate as 'No' if 30.1 is 'No'

© 2020 Prager R et al. *JAMA Network Open.*

eTable 3: Complete List of Included Studies With STARD Adherence

First Author	Journal	Country	STARD Items Reported (total = 30)	Pubmed ID
Akoglu	American Journal of Emergency Medicine	Turkey	19.7	29146418
Aksay	American Journal of Emergency Medicine	Turkey	15.8	26851063
Avci	American Journal of Emergency Medicine	Turkey	16.1	27645809
Aykol	American Journal of Emergency Medicine	Turkey	21.7	26935225
Bergmann	Critical Care	Germany	21.2	29409525
Caglar	American Journal of Emergency Medicine	Turkey	16.3	28552270
Calder	Journal of Trauma and Acute Care Surgery	USA	14.3	28590347
Christian	Journal of Trauma and Acute Care Surgery	USA	20.7	29794689
Cowie	European Journal of Anaesthesiology	Australia	18.3	29194227
Cowie	European Journal of Anaesthesiology	Australia	15.9	26225501
Daley	American Journal of Emergency Medicine	USA	22.1	27793505
Daniels	Annals of Emergency Medicine	USA	21.7	26747219
Daswani	Academic Emergency Medicine	USA	23.8	27155438
Doniger	Pediatric Emergency Care	USA	20.5	27299296
Doniger	Pediatric Emergency Care	USA	18.4	27299294
Dwyer	American Journal of Emergency Medicine	USA	19.2	29174452
Filopei	Critical Care Medicine	USA	19.3	28953498
Garcia	The Journal of Emergency Medicine	Spain	20.2	29306580
Gil-Juanmiquel	Pediatric Critical Care Medicine	Spain	18.3	27801709
Gungor	Academic Emergency Medicine	Turkey	20.0	28171688
Gungor	American Journal of Emergency Medicine	Turkey	17.8	27358042
Helland	Academic Emergency Medicine	USA	21.2	27428394
Jay	European Journal of Anaesthesiology	France	24.2	27259094
Jones	Pediatric Emergency Care	United Kingdom	21.0	28509686
Karacabey	American Journal of Emergency Medicine	Turkey	15.9	26994679
Kilic	American Journal of Emergency Medicine	Turkey	14.6	27292603
Kim	The Journal of Emergency Medicine	Canada	21.2	27637139
Kinnaman	The Journal of Emergency Medicine	USA	22.8	26830361
Kocaoglu	American Journal of Emergency Medicine	Turkey	16.2	27396537
Kozaci	Injury	Turkey	17.5	28069140
Kozaci	Injury	Turkey	17.0	28431818
Kumar	Journal of Cardiothoracic and Vascular Anesthesia	India	16.2	28826847
Kwon	American Journal of Emergency Medicine	Republic of Korea	18.3	27339224
Lahham	American Journal of Emergency Medicine	USA	21.2	29162442
Lau	American Journal of Emergency Medicine	Hong Kong	19.1	29162443
Laursen	Scandinavian Journal of Trauma, Resuscitation and Emergency Medicine	Denmark	19.3	27480128
Lee	Injury	Republic of Korea	22.7	28729007
Lee	American Journal of Emergency Medicine	Republic of Korea	18.9	29307765
Lin	Pediatric Emergency Care	USA	19.1	28072673
Liu	CHEST	China	18.8	26836942

© 2020 Prager R et al. *JAMA Network Open.*

Liu	American Journal of Emergency Medicine	China	15.7	28404216
Liu	American Journal of Emergency medicine	China	15.4	26614584
Marchese	Pediatric Emergency Care	USA	20.4	28146012
McCormick	Pediatric Emergency Care	USA	16.9	26308609
Mohammadrezaei	American Journal of Emergency Medicine	Iran	19.2	28185748
Mongodi	CHEST	France	23.1	26836896
Moser	British Journal of Anaesthesia	Canada	19.6	29028915
Nazerian	Academic Emergency Medicine	Italy	25.8	27859891
Oguz	American Journal of Emergency Medicine	Turkey	18.5	28139307
Ozturk	American Journal of Emergency Medicine	Turkey	19.3	28807445
Poonai	Academic Emergency Medicine	Canada	24.6	27976448
Pujol	American Journal of Emergency Medicine	France	20.4	29653786
Raffiz	American Journal of Emergency Medicine	Malaysia	18.2	27852525
Ramsingh	Anesthesiology	USA	21.8	26950708
Rowlands	The Journal of Emergency Medicine	Australia	25.8	27814988
Russell	The Journal of Emergency Medicine	USA	21.6	28285867
Samson	European Journal of Emergency Medicine	Spain	21.4	27536810
Sartini	European Journal of Emergency Medicine	England	19.7	26891086
Sauter	Emergency Medicine Journal	Switzerland	21.1	28500086
Scharonow	Scandinavian Journal of Trauma, Resuscitation and Emergency Medicine	Germany	13.8	29914554
Schellenberg	Journal of Trauma and Acute Care Surgery	USA	16.5	29958248
Sen	Journal of Critical Care	USA	22.2	28342384
Sullivan	American Journal of Emergency Medicine	USA	16.7	27209300
Topin	The Journal of Emergency Medicine	France	20.2	26972017
Touw	Anaestheisa	Netherlands	19.9	29529332
Varshney	Emergency Medicine Journal	Canada	24.7	27107052
Weekes	Annals of Emergency Medicine	USA	25.0	26973178
Williamson	Neurocritical Care	USA	22.0	26209281
Wong	American Journal of Emergency Medicine	Hong Kong	18.6	28038826
Zanobetti	CHEST	Italy	18.3	28212836
Zanobetti	European Journal of Trauma and Emergency Surgery	Italy	17.0	26683569
Zhan	Pediatric Emergency Care	Denmark	21.0	27749801
Zieleskiewicz	British Journal of Anaesthesia	France	25.5	27440631
Zieleskiewicz	Injury	France	18.9	30017184

eTable 4. Subgroup Analysis of STARD Adherence by Study Design

**Study Design**	**Number of Studies**	**Mean STARD Items ± SD**
Prospective	68	19.7 ± 2.9
Retrospective	6	19.7 ± 1.8
Welch's two-sample t-test p > 0.99		

© 2020 Prager R et al. *JAMA Network Open.*

eTable 5. Subgroup Analysis of STARD Adherence by Country[a]

Country[b]	Number of Studies	Mean STARD Items ± SD
Australia	3	20.0 ± 4.2
Canada	4	22.5 ± 2.2
China	3	16.6 ± 1.5
Denmark	2	20.1 ± 0.9
France	6	22.1 ± 2.4
Germany	2	17.5 ± 3.7
Hong Kong	2	18.8 ± 0.2
Italy	3	20.4 ± 3.9
Republic of Korea	3	20.0 ± 1.9
Spain	3	20.0 ± 1.3
Turkey	14	17.6 ± 1.9
USA	22	20.3 ± 2.5
Other	7	19.3 ± 1.6

a)Top 10 countries ranked by number of included studies published by corresponding authors from that country; 12 were included because three were tied for 10th. Countries not in the top 10 were classified as “other
ANOVA p = 0.023
A Tukey honest significant difference test showed a difference France was compared to Turkey (22.1 vs 17.6, p = 0.043).

eTable 6. Subgroup Analysis of STARD Adherence by Body System

Body Area	Number of studies (N = 74)	Mean STARD items ± SD
Abdominal	16	20.0 ± 2.5
Head and Neck	6	17.8 ± 1.6
MSK	16	19.2 ± 3.1
Thoracic	31	20.2 ± 2.8
Other/Procedural	5	19.8 ± 2.7

ANOVA p = 0.29

eTable 7. Subgroup Analysis by STARD Adoption by Journal

STARD Adopters	Number of studies (N = 74)	Mean STARD items ± SD
Yes	41	20.5 ± 2.9
No	33	18.6 ± 2.3

Welch’s two-sided t-test p = 0.002

eTable 8. Subgroup Analysis by STARD Citation in Article

STARD Citation	Number of studies (N = 74)	Mean STARD items ± SD
Yes	5	21.3 ± 0.9
No	69	19.5 ± 2.9

Welch’s two-sided t-test p = 0.013

eTable 9. Subgroup Analysis by Patient Population (Adult vs Pediatric vs Mixed)

Population[a]	Number of studies (N = 71)	Mean STARD items ± SD
Adult	44	20.0 ± 2.7
Pediatric	17	20.2 ± 3.1
Mixed	10	17.9 ± 1.9

a) 3 studies did not report population
ANVOA p = 0. 085

© 2020 Prager R et al. *JAMA Network Open.*

eTable 10. Subgroup Analysis of the Five Most Common Journals

Journal	Number of Studies (N = 46)	Mean STARD Items ± SD
Academic Emergency Medicine	5	23.1 ± 2.2
American Journal of Emergency Medicine	24	18.1 ± 2.1
The Journal of Emergency Medicine	6	22.0 ± 1.9
Pediatric Emergency Care	7	19.6 ± 1.4
Injury	4	19.0 ± 2.2
ANOVA p < 0.001<br>‘Academic Emergency Medicine’ and ‘The Journal of Emergency Medicine’ had a significantly higher number of reported items compared to the ‘American Journal of Emergency Medicine’ (23.1 and 22.0 vs 18.1, p = 0.002 and p = 0.018, respectively)		

eTable 11. Subgroup Analysis by Use of Supplemental Materials

Supplemental Material Included	Number of studies (N = 74)	Mean STARD items ± SD
Yes	8	19.2 ± 3.0
No	66	19.7 ± 2.8
Welch’s two-sided t-test p = 0.91		

eTable 12. Subgroup Analysis by Impact Factor (Median Split)

Impact Factor (median split)	Number of studies (N = 74)	Mean STARD items ± SD
> 1.65	35	20.3 ± 3.2
≤ 1.65	39	19.1 ± 2.3
Welch’s two-sided t-test p = 0.078		

© 2020 Prager R et al. *JAMA Network Open.*
